# Supplementary material for: Phantom touch illusion, an unexpected phenomenological effect of tactile gating in the absence of tactile stimulation
Source: Sci Rep. 2023 Sep 18;13:15453. doi: 10.1038/s41598-023-42683-0 (PMC10507094; doi:10.1038/s41598-023-42683-0)
Supplement: Supplementary file 1 — Supplementary Information. [file 41598_2023_42683_MOESM1_ESM.pdf]

Supplementary materials

Supplementary figure 1

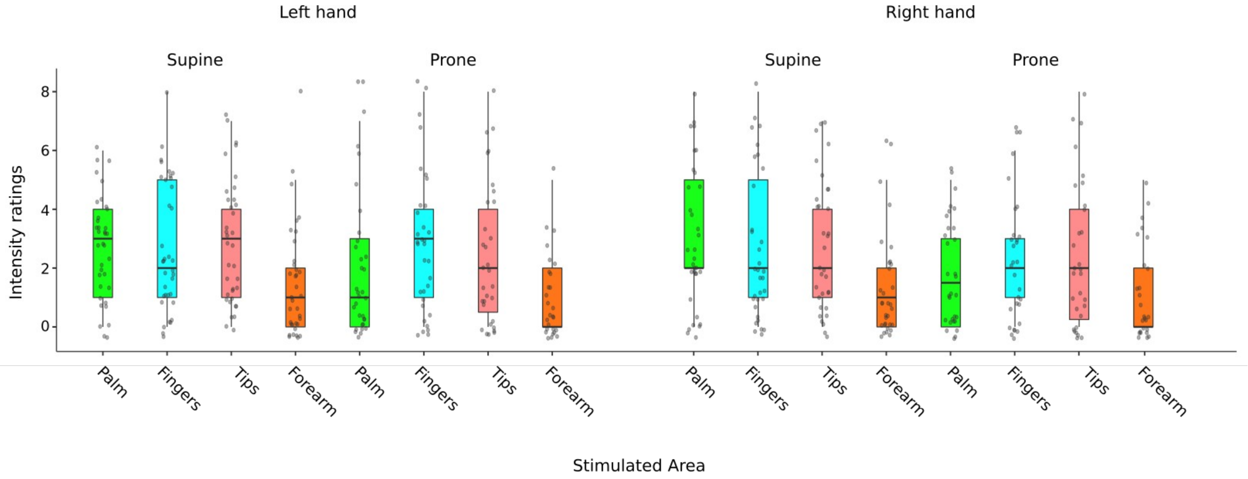

Left- and right-hand results of phantom touch intensity ratings across different locations on the hand and the forearm. Box colors represent the relevant parts of the hand as depicted on the right; horizontal line is the median; gray dots represent individual data points; whiskers represent 1.5 times the interquartile range.

Supplementary Table 1

Individual subjects’ ratings of PTI intensity, subject data, specific verbal descriptions and their classifications.

Subjects	Left								Right								[illegible]h	Moving	Gender	Subject statements	Classification
	Supine				Prone				Supine				Prone								
1	2	2	2	2	2	2	2	2	2	2	2	2	2	2	2	2	1	1	1	Like touching soap bubbles/static	Tingling
2	3	2	3	5	0	0	0		4	2	3	6	3	3	3		1	1	0	Tingling / tickling	Tingling
3	1	1	1	1	1	1	1	1										1	1	Thought I am using stimulator	Tingling
4	1	1	1	1														1	0	Tingling (formigamento)	Tingling
5	2	2	2	4														0	0	Warm feeling, like wood texture	Warm
6	4	2	2		3	3			3	2	2	0				0		1	0	Tingling (formigamento)	Tingling
7	3	5	3	0	6	7	6	0	6	6	6	0						1	0	Tingling (formigamento)	Tingling
8	2	1	1	1					2	1	1							1	0	Tickle	Tingling
9	3	5	5	0	0	5			0	0	0	0	0	0	0			1	1	Like wind going through the hand	Airflow
10	1	1	1	0	1	1	1	0	1	1	1	0	1	1	1	0		1	1		Tingling
11	0	0	0	0	0	0	0	0	0	0	0	0	0	0	0	0	1	0	0		
12	5	4	7	2	0	3	5		5	6	7	1	1	5	7		1	1	1	Like bubble of soap	Foam
13	4	6	6	5	8	8	8		5	6	6	0	4	7	7		0	1	0	Like air coming through the hand	Airflow
14	3	3	4	2	3	3	3		2	2	2	1	2	2	2		0	1	0	Like air coming through the hand	Airflow
15	4	6	5	1	7	7	7	1	5	7	7	5	5	7	8	3	1	1	0	It is like a little wind	Airflow
16	4	6	4	2	6	3	1	0	2	2	0	2	3	2	0	4	1	1	0	Like the stick touch, it is soft like velvet	Velvet
17	3	2	2	2	0	1	1	2	3	1	1	1	0	1	1	1	1	1	0	Slihtly cold feeling, as if the hairs on palm and forearm raises	Tingling
18	3	2	1	4	4	3	1	3	3	2	1	3	3	3	1	3		1	1	mostly prickling, on pinky fingers and forearm warm feeling	Tingling
19	1	1	1	1	2	1	0	0	3	1	1	1	0	1	0	0		1	1	on hand: tickle; on forearm: weak pressure	Tingling
20	6	5	7	3	3	4	3	1	7	7	5	3	5	7	6	3		1	0	on hand: like striking the hair; fingertips had warm feeling	Tingling
21	2	2	4	2	1	4	1	2	4	5	5	0	2	2	3	0		1	1	sensation: tickle	Tingling
22	5	5	4	2	1	3	2	3	7	3	7	4	5	3	2	0	1	1	0	right hand); Feeling the stick to	Cold
23	3	5	5	0	2	5	4	0	6	3	3	1	4	4	5	1	1	1	1	ack hand - Pressure; left forearm	Tingling
24	0	0	0	0	0	0	0	0	0	0	3	0	0	0	2	0	0	1	1		Tingling
25	1	0	1	0	1	2	0	0	2	1	2	0	1	1	2	1	1	1	0	ne l) When touching the forearm	Airflow
26	4	2	3	0	0	0	0	0	8	7	4	6	3	6	1	5	1	1	0	rm feeling (left); Cold feeling (rig	Warm
27	3	0	3	3	2	3	4	2	0	0	3	1	0	3	0	0	1	0	0	0	
28	2	1	1	0	1	1	2	1	2	1	1	1	1	1	1	0	1	1	0	"Feeling my arm" (prone forear	Other
29	3	4	3	0	0	8	5	0	0	0	0	0	0	0	3	0	0	1	1	something very real touching my	Touch
30	0	0	0	0	0	0	0	0	0	0	0	0	0	0	0	0	0	0	0		
31	6	8	6	8	8	4	7	5	7	8	4	2	4	3	5	4	1	0	1	Touch	Touch
32	6	5	6	3	5	5	6	3	5	5	5	2	4	4	5	2	1	1	0	Pressure	Pressure
33	4	2	4	0	1	2	3	0	2	3	4	0	3	1	4	0	1	1	0	Tingly	Tingling
34	1	1	1	0	0	0	1	0	2	1	1	0	0	0	0	0	1	1	0	t supine fingertips); Pressure (lef	Tingling
35	0	0	0	0	0	0	0	0	0	0	0	0	0	0	0	0	0		1		
36	3	5	3	1	0	3	3	1	2	3	4	1	0	3	4	1	1	1	1	pressure, tingly (right supine fo	Tingling

Supplement 1

Control experiment

Methods

Subjects

Thirty-two volunteers (fifteen males), all right-handed participated in the experiment. Fourteen of these subjects also participated in the main VR experiment (the order of main vs. control experiments was randomized and counterbalanced across subjects). All of the subjects were naiive to the experimental hypotheses and had none or little prior experience with VR. All procedures were approved by the local ethics committee of the Ruhr-University Bochum and were performed in accordance with the declaration of Helsinki. Subjects provided an informed consent prior to the beginning of the experiment.

Procedures

The procedures mirrored those from the main experiment except from that the subjects were not using a virtual reality headset and, instead of a virtual stick, they held a small laser pointer in their right hand. The laser pointer could project a small (ca. 5mm diameter) point of red light onto the skin of participant's left hand. There were two conditions: “Laser on”, in which we asked the subjects to stroke their hand with the laser point of light and “Laser off”, in which we asked the subjects to move the pointer as if they were stroking their hand with the point of light. These conditions aimed to test subjects' tendency to 1) report “tingling” in the case a visual stimulation was delivered to the hand previously demonstrated to produce thermal sensation [1] or 2) report tactile stimulation in the case no visual stimulation was delivered to the hand but hand was merely attended, in a way similar to reported by Cataldo et al. [2]. The condition order was counter-balanced and randomized across participants.

Results

In the “Laser on” condition, 29 out of 32 subjects reported having either a thermal or a tactile illusion, in accordance with previous studies. Of these, 13 reported feeling “warmth” and 4 “tingling” where the laser spot illuminated their skin (See Supplementary Figure 2). In contrast, in the “Laser off” condition, 14 out of 32 subjects reported feeling any sensation, without a clearly dominant quality with just 3 subjects reporting “tingling” (Supplementary Figure 2). Results of the proportion test indicated that there is a significant difference between “Laser off” and “Laser on” in number of reported perceptions $Z = 3.99$, $p < .001$.

References

[1] Michel, C., Velasco, C., Salgado-Montejo, A., & Spence, C. (2014). The Butcher's Tongue Illusion. *Perception*, *43*(8), 818–824. https://doi.org/10.1068/p7733

[2] Cataldo, A., Di Luca, M., Deroy, O., & Hayward, V. (2023). Touching with the eyes: oculomotor self-touch induces illusory body ownership. iScience, 26(3), [106180].

Supplementary Figure 2

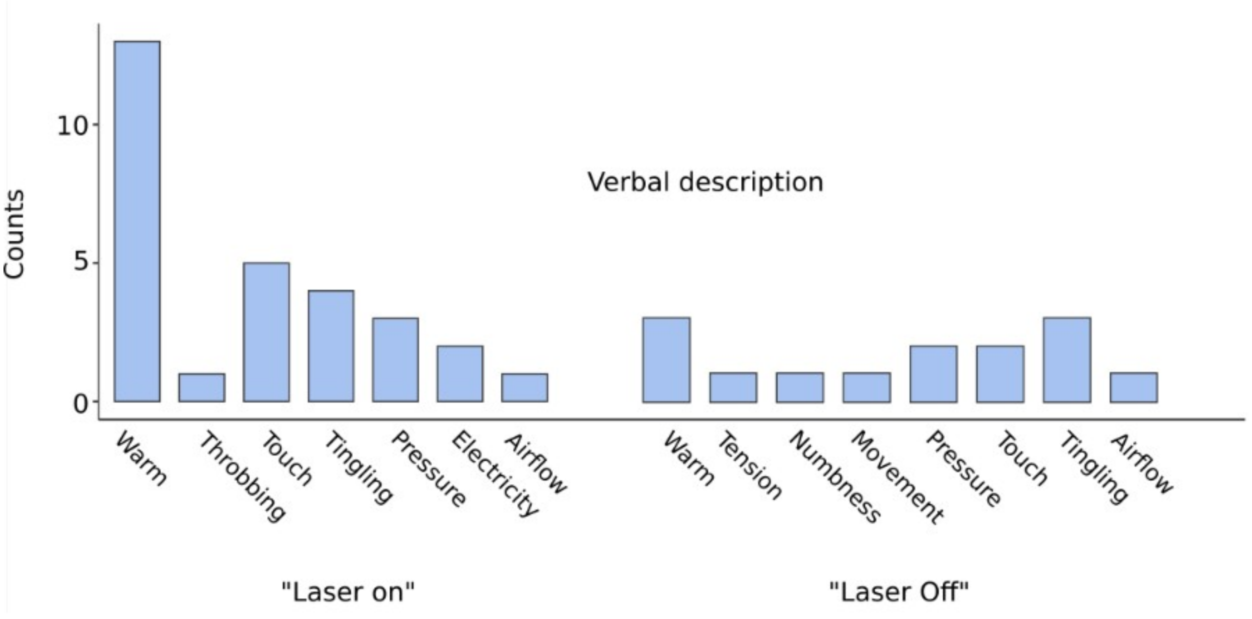

Frequencies of verbal descriptions of the skin sensations in the control experiment for “Laser On” and “Laser Off” conditions.
